# Pattern of BOLD signal in auditory cortex relates acoustic response to perceptual streaming

**DOI:** 10.1186/1471-2202-12-85

**Published:** 2011-08-17

**Authors:** Kevin T Hill, Christopher W Bishop, Deepak Yadav, Lee M Miller

**Affiliations:** 1 Center for Mind and Brain, University of California, 267 Cousteau Pl, Davis, California, 95616, USA; 2 Department of Neurobiology, Physiology and Behavior, University of California, One Shields Ave, Davis, California, 95616, USA

## Abstract

**Background:**

Segregating auditory scenes into distinct objects or streams is one of our brain's greatest perceptual challenges. Streaming has classically been studied with bistable sound stimuli, perceived alternately as a single group or two separate groups. Throughout the last decade different methodologies have yielded inconsistent evidence about the role of auditory cortex in the maintenance of streams. In particular, studies using functional magnetic resonance imaging (fMRI) have been unable to show persistent activity within auditory cortex (AC) that distinguishes between perceptual states.

**Results:**

We use bistable stimuli, an explicit perceptual categorization task, and a focused region of interest (ROI) analysis to demonstrate an effect of perceptual state within AC. We find that AC has more activity when listeners perceive the split percept rather than the grouped percept. In addition, within this ROI the pattern of acoustic response across voxels is significantly correlated with the pattern of perceptual modulation. In a whole-brain exploratory test, we corroborate previous work showing an effect of perceptual state in the intraparietal sulcus.

**Conclusions:**

Our results show that the maintenance of auditory streams is reflected in AC activity, directly relating sound responses to perception, and that perceptual state is further represented in multiple, higher level cortical regions.

## Background

The natural world presents a rich mixture of auditory events that overlap in frequency and time. One of the brain's greatest perceptual challenges is to segregate this mixture into distinct "streams", so that it can attribute acoustic energy to discrete sources in the environment. This analysis of an auditory scene is essential for much of our daily acoustic experience, notably for communication where it is posed as the 'cocktail party problem' [1]. In addition to its importance for healthy listeners, stream segregation may be impaired in various neurological disorders such as dyslexia [2], schizophrenia [3] and Asperger syndrome [4], and the inability to segment and selectively attend to sounds is a major problem with hearing impairment [5,6].

Decades of psychoacoustic studies have characterized the basic phenomenology of streaming with sequences of sounds. The classic paradigm uses alternation between two sounds that differ along one stimulus dimension [7-9], such as spatial location [10,11]. The sounds (usually referred to as A and B) typically alternate along with silent gaps (-) in an ABA- pattern. When these stimuli are close in the relevant dimension they are grouped into a single stream and perceived as triplets with a galloping rhythm. At larger separations the streams segment, and subjects perceive a repeating stream of A sounds (A-A-A-) and a separate, more slowly repeating B stream (B---B---). At intermediate frequency separations the single and two stream percepts are bistable, where listeners switch between perceptual states after an initial buildup [12,13]. However, despite its perceptual importance, the neural mechanisms of streaming remain unclear.

A central area of contention is the role of early auditory cortex in forming and maintaining streams [14]. Evidence from different methodologies has failed to converge on a single answer. Animal studies have relied mainly on recordings from early auditory cortex that characterize the changing neural representation of tones during the buildup of streaming [15] or physical changes to the stimulus that correlate with perceptual state [16,17]. Theories based on this data posit that auditory cortex (AC) plays a key role in both the formation and maintenance of auditory streams through modulation of the receptive fields of auditory neurons [18]. However these conclusions are practically limited since it is difficult to record extracellularly in many regions of cortex simultaneously and since animals cannot signal their perceptual state unambiguously. Meanwhile, human studies using both electroencephalography (EEG) [19,20] and magnetoencephalography (MEG) [21,22] have also supported the importance of AC in streaming. These studies found correlates of segregation in electrical and magnetic waveforms believed to be generated in AC and time locked to the individual tones within a sequence. However the stimulus-locked nature of waveform analysis could not characterize non-AC signals which occur on the time scale of percepts rather than individual sounds.

In contrast, an influential fMRI study by Cusack in 2005 challenged the importance of AC by showing a single area in right posterior IPS where activity was greater during the split percept relative to the grouped percept [23], and failing to find any effect of percept in AC. These findings led Cusack to propose a model of stream segregation that relied on top-down control of auditory information for the maintenance of streams rather than automatic segregation in early sensory cortex. He argued that IPS is a multimodal region sensitive to object number and provides the key neural mechanism for the segmentation of auditory sources. Finally, recent fMRI experiments have found effects related to streaming in AC, either as stimulus properties change in a way that correlates with streaming [24,25] or during the momentary switches from one percept to another [26,27]. However, it is unclear how these stimulus driven effects or switch events are related to the persistent neural activity that maintains a single percept over an extended period of time.

Taken as a whole, these findings from multiple methodologies present an inconsistent picture of the neural mechanisms of auditory streaming. Animal researchers have clear theories for the neural mechanisms in AC that could sustain streaming, but have thus far not recorded from cortical regions outside of auditory cortex. EEG and MEG evidence suggests an involvement of AC in the continuous maintenance of auditory streams yet fMRI experiments have failed to find corroborating evidence. Therefore, in this study, we used fMRI to provide some continuity between disparate lines of evidence: specifically, to test if the same networks that strongly represent incoming auditory information are sensitive to perceptual state by showing an overall activity difference between group and split percepts. We observe an effect in early AC which is sustained through the length of the percept.

## Methods

### Participants

Fifteen subjects participated in the study (mean age 22.1 years +/- 1.8 years SD). All subjects had no history of neurological disorders or hearing loss. Participants gave written informed consent in accordance with procedures approved by the University of California Institutional Review Board and were paid for their participation. Two of the subjects were removed based on behavioral performance inside the scanner (see results section).

### Stimulus Design

**Figure 1:**
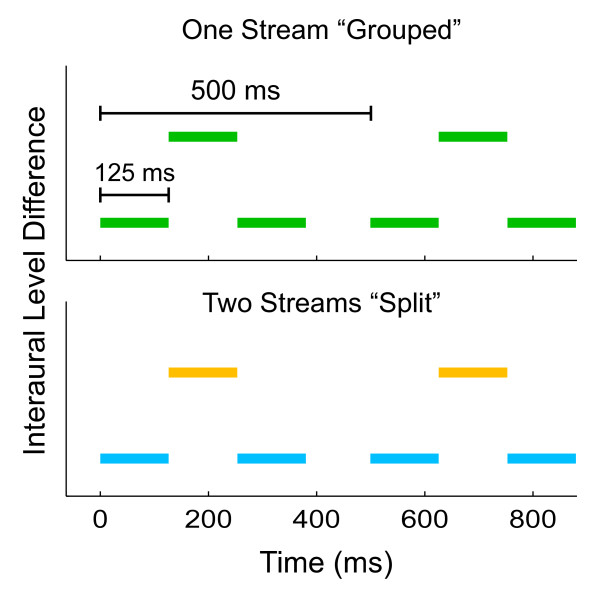
**Stimulus diagram**. A diagram of stimulus parameters that shows two triplets. The length between each triplet was 500 ms, with 125 ms stimulus onset asynchrony (SOA) between each tone. Tones were separated by interaural level difference (ILD). The coloring reflects two possible perceptions of an identical tone sequence. Above, the single stream or grouped percept has all tones as part of the same perceptual object. Below, the two-stream or split percept has tones with different features grouped into different perceptual objects. Two types of tone sequences were presented that were either perceived as *left-right-left *or *right-left-right *sequences.

The stimulus consisted of repeating sequences of two sets of harmonic pitch sounds (A and B) and gaps (-), presented binaurally at a comfortable level, approximately 80 dB. The sounds were arranged in an ABA- pattern. Each of the complex tones in the ABA triplets had a stimulus onset asynchrony (SOA) of 125 ms, with a 10 ms linear ramp. The long gap (-) had a duration of 125 ms to ensure that the sounds in segmented A and B streams were isochronous (Figure 1). This arrangement of tones is known to induce streaming when the A and B sounds are separated along some stimulus dimension. For this study, the A and B sounds were separated in perceived spatial location using interaural level difference (ILD). The ILD was calibrated for each subject during a pre-scan session targeting 50% of the time spent streaming (mean 5.85 dB, +/- 2.10 SD). In different versions of this basic ABA- pattern, the A tone was alternatively perceived on the left or right side of the midline. The B tone was always perceived on the opposite side of the midline from the A tone with equal spatial disparity. This gives two sequences: a *left-right-left-gap *(LRL) and a *right-left-right-gap *(RLR). Both the A and B sounds had a fundamental frequency of 180 Hz and equal intensity harmonic stacks up to 16 kHz. The fundamental frequency was chosen to be in the range of the human voice, a highly ecologically relevant stimulus subject to streaming. Each stimulus block consisted of either a LRL or RLR sequence continuously for 100 seconds. When outside of the fMRI scanner, a recording of the scanner's EPI noise was presented with the ABA- triplets to ensure accurate estimation of behavioral thresholds. The EPI noise was recorded using an Optimic 1150 optical microphone (Opoacoustics http://www.optoacoustics.com/ and adjusted in the sound files to the sound level and signal-to-noise ratio that would be experienced by the subjects in the fMRI scanner.

### Intensity Deviants

In addition to the basic streaming stimuli, we included increased intensity deviants for both A and B sounds to ensure that subjects were actively attending to the sound sequences, and so we could confirm that detection - as a proxy measure of attention - does not explain any perceptual streaming effects. Deviants occurred 10 times for each sound at pseudo-random times throughout the 100 second block. The deviants were also calibrated for each subject to target a *d' *of 2 (group mean 2.77 dB, +/- 1.00 SD).

### Task and Calibration

Subjects were given a short training on how to distinguish the two percepts, which typically alternated spontaneously and categorically between a single stream containing both the A and B tones (referred to as *grouped*) and two separate streams of the A tones alone and the B tones alone (referred to as *split*). Once they were comfortable with the distinction, subjects began the full calibration. First, a rough psychometric function for streaming as a function of ILD separation was calculated using three 100 second long blocks. Subjects were instructed to press and release one of two buttons with their right middle and right index fingers to indicate a switch to a grouped and split percept, respectively. All subjects were assumed to begin each block in the *grouped *percept [12]. Subjects were given a self-timed break between each 100 sec block. Streaming thresholds, defined as the spatial disparity necessary for the subjects to spend 50% of a block in the split percept, were estimated from each subject's approximate three-point psychometric function through linear interpolation. Then, with spatial disparity held at this threshold, detection thresholds for the intensity deviants were estimated in a similar fashion. Subjects pressed a button with their left index finger each time they detected a deviant. Responses occurring from 200-1200 msec after the onset of the deviant were scored as hits; responses outside of this window were counted as false alarms. To further refine our estimate of each subject's ILD and deviant detection thresholds, subjects then began an adaptive 1-up 1-down staircase procedure [28], with the initial values set at the previously estimated thresholds. This algorithm targeted 50% of the time spent in the split percept during each 100 sec block, and a *d' *of 2 for deviant detection across both deviant types. Subjects performed both tasks simultaneously, and after each block the values for spatial separation and deviant disparity were adapted independently. The spatial separation had a step size of 1 dB and the detection task had a step size of 0.5 dB. Subjects proceeded until the direction of change reversed at least 8 times for each of the two metrics, a procedure that usually lasted 10-14 blocks. Then, the values for each reversal were averaged to find a threshold for streaming and deviant detection. This threshold was finally confirmed with a 6 block run, and adjusted by hand if the average streaming differed from 50% by more than 10%, and if the *d' *was less than 1.5 or more than 3. This calibration procedure occurred within two weeks before a participant's scanning session, and lasted approximately 1.5 hours.

### Scanning procedure

Scanning was separated into six, 8.47 minute sessions. Sessions began and ended with a 30 second fixation period that served as a baseline. Subjects performed the previously described streaming and deviant detection tasks during four, 100 sec blocks each separated by a 16 sec fixation period. Each session consisted of 2 LRL and 2 RLR stimulus blocks in pseudo-random order.

### Imaging

MRI data were collected in a Siemens MAGNETOM Tim Trio System 3 Tesla scanner with a 32-channel RF headcoil and a whole body gradient system. Foam padding was used to minimize head motion. Each session began with a series of images to determine regional anatomy, including a sagittal localizer (Repetition Time (TR) = 250 ms, Echo Time (TE) = 3.2 ms). Single-shot gradient-echo echoplanar images (EPI) were acquired for thirty-six near-axial slices. The functional scans had the parameters: TR of 2 s, TE 25 ms, 64 × 64 acquisition matrix, 3.4 mm slice thickness, a 220 mm field of view, 3.45 mm in plane resolution, bandwidth of 2604 Hz/Px and a flip angle of 90°. A high resolution three-dimensional MPRAGE image for use in intersubject coregistration was taken at the end of the session with a voxel size of 0.45 × 0.45 × 0.95 mm. Auditory stimuli were presented with a piezoelectric audio system customized for use in high magnetic fields (Sensimetrics Corp, model S14 http://www.sens.com/s14/). The earbuds of the audio system passively attenuated the scanner noise to 60 dB (attenuation level based on the manufacturer's specifications), and stimuli were played at 80 dB. All sounds were filtered to account for known frequency response of the earbuds, ensuring that stimuli were perceived as intended.

### Data Analysis

Behavioral data was analyzed using custom in-house scripts written in Matlab 7.4 (Mathworks, http://www.mathworks.com/). fMRI data was analyzed using a combination of in-house scripts and the modified general linear model (GLM) in SPM 8 http://www.fil.ion.ucl.ac.uk/spm/software/spm8/. EPIs were slice time corrected, realigned to the first scan, coregistered to the subject's MPRAGE, normalized to the Montreal Neurological Institute (MNI) template [29], and smoothed with an 8 mm Gaussian smoothing kernel unless otherwise noted. The following covariates were added to the design matrix: a block regressor for the 100 second sound sequences (one for each type of stimulus, LRL and RLR), a perceptual regressor which had the value of 1 when subjects grouped sounds and -1 when subjects split sounds, and separate impulse regressors for deviant onsets, hits and false alarms. The standard approach in neuroimaging studies would be to model the key conditions (grouped and split) separately, and then contrast their parameter estimates or betas. For bistable perceptions this standard method poses significant problems. The categorical nature of the perceptual phenomenon requires that when one of the two possible states ends, the other begins. Thus after standard high-pass filtering, perceptual states are strongly collinear (with a correlation coefficient more extreme than -0.90 in our tests). Models with highly collinear regressors are mathematically unstable and can lead to unreliable results [30]. The inclusion of a bimodal covariate to model perception surmounts this limitation by combining the strongly-anticorrelated regressors into a single covariate. This bimodal regressor is functionally equivalent to a grouped > split contrast between the parameter estimates of independent regressors. Therefore, positive regression coefficients associated with the perceptual regressor signals regions which have higher activity levels during grouped percept relative to split percept, while negative parameter estimates indicate the inverse. All these regressors were convolved with the standard SPM8 hemodynamic response function (HRF). Motion parameters and session covariates were also included as nuisance regressors. All group level statistical tests were t-tests on beta parameter estimates against the null hypothesis that they equal zero.

## Results

### Behavior

In order to maintain statistical independence between regressors used to code sound onset and perceptual state, only subjects who streamed between 35% and 65% during the scanning session were used. Subjects with larger or smaller streaming percentage would by definition have large portions of sound blocks spent in a single percept, which would cause the sound onset and percept to have similar time courses, leading to collinear regressors. Thirteen of the 15 subjects met this criterion, and represent the group referred to in all subsequent analyses. Within this group, the mean proportion of streaming was 49.5% (+/- 8.0% SD). There was no significant difference in streaming percentage between the two stimulus types, LRL and RLR and all subsequent analyses are collapsed across both stimulus types. The group mean of each subject's median inter-switch interval was 9.3 seconds (+/- 4.3 seconds SD), putting it in a range which is amenable to detection by a GLM after filtering with a HRF.

The mean *d' *for deviant detection was 1.84 (+/- 0.9 SD), indicating that subjects actively attended to the stimuli. To further analyze the effect of perceptual state on deviant detection, we performed an analysis of variance (ANOVA) on the target hit rate in a repeated measures 2 × 2 design, with the factors of deviant side and perceptual state. Hit rate was used for the ANOVA because false alarms cannot be attributed to a particular side. This ANOVA revealed that there was a main effect of target location on hit rate (F_(1,12) _= 8.23, p = 0.014), with left targets being detected with more frequency than right targets. There was no effect of perceptual state (F_(1,12) _= 0.37, p = 0.55) or interaction between location and perceptual state (F_(1,12) _= 1.31, p = 0.27).

### fMRI

**Figure 2:**
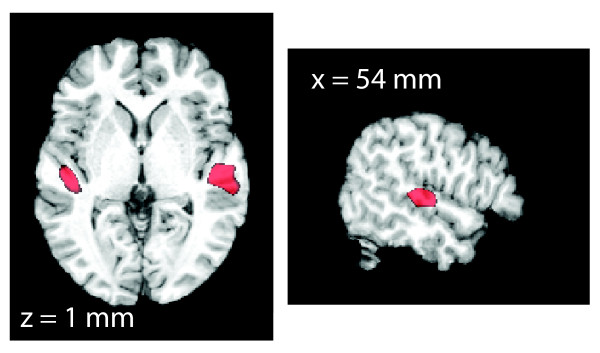
**Auditory cortex region of interest**. The voxels stongly activated by sound sequences along the superior temporal gyrus are shown in red. Significance threshold was corrected for multiple comparisons by Bonferroni correction. Activity in this ROI showed a significant effect of perceptual state (p < .05). The average beta across the ROI and subjects is negative, indicating that the split percept results in higher levels of activation within AC than during the grouped percept.

When testing for an effect of the sound covariate relative to baseline, we observe robust activations along portions of superior temporal gyrus (STG) and brainstem. Only auditory cortex (AC) and inferior colliculus were found to be significantly modulated when the conservative Bonferroni family wise error (FWE) correction for multiple comparisons was used (p < 0.05). The cortical activation likely contains both primary and secondary auditory regions, and our paradigm was not meant to distinguish between them. To test the direct hypothesis that perceptual state is encoded by regions that process attributes of the stimulus, we used only those voxels passing FWE correction bilaterally along the superior temporal gyrus as a region of interest (ROI) (Figure 2). We averaged all parameter estimates, or betas, within the ROI and tested for a non-zero beta for our streaming regressor across all subjects. A positive beta would indicate that activity in those voxels is higher for the grouped percept than the split percept, while a negative beta would signify the inverse. For more details about the perceptual regressor, see the Methods section. We find a significant effect of percept in voxels that are strongly responsive to sound (p = 0.035). The average beta across the ROI and subjects is negative, indicating that the split percept results in higher levels of activation within AC than during the grouped percept. A similar test on the voxels within the inferior colliculus yielded no effect of percept. However, our scanning procedure was not optimized for detection of subcortical signals [31].

**Figure 3:**
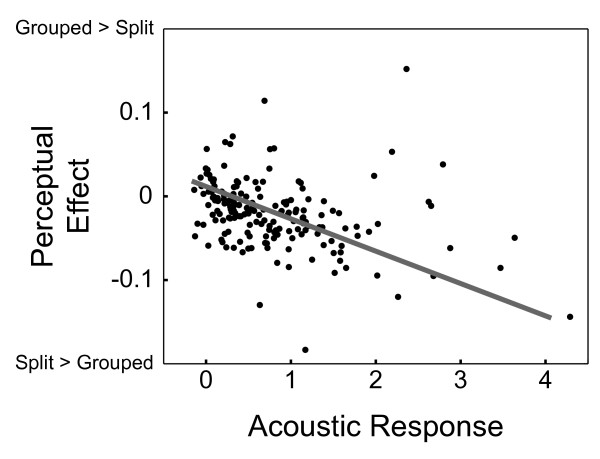
**Voxels within auditory cortex sensitive to auditory stimuli are also sensitive to perceptual state**. The pattern of voxel by voxel activity levels within the AC ROI reveals a consistent relationship between the strengh of the auditory response and the effect of percept. Shown here is data from the subject with median correlation value between the beta for sound and the beta for the perceptual regressor. When analyzing the correlation values across the entire subject group, the z-scores of these correlation values were significantly negative (p < .05), indicating that voxels with a stronger level of activation for sound also had a stronger effect of percept, with the split percept causing greater activity than the grouped percept. Each point represents a single voxel within the ROI, and axis values indicate coefficients obtained from the GLM (arbitrary units).

In addition, the spatial pattern of perceptual modulation and the representation of sound within AC covaried on a voxel by voxel basis. Using the same AC ROI, we analyzed the relationship between beta values for the sound regressors and the perceptual regressors in unsmoothed data. For each subject, correlation coefficients were transformed to z-scores using the hyperbolic arctangent, and then group z-scores were tested against the null hypothesis. This analysis indicated that there was a significant (p < .05) negative correlation between the beta values, indicating that those voxels which had a stronger level of activation for sound also had a stronger effect of percept, where the split percept caused greater activity than the grouped percept. The mean z-score across the group corresponded to a correlation coefficient of -0.31. The data for the subject with the median correlation coefficient (-0.32) is presented in Figure 3.

**Figure 4:**
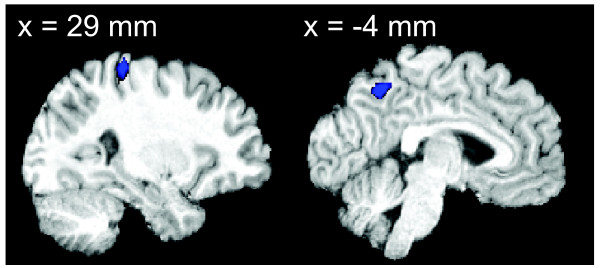
**Effects of percept outside auditory cortex**. A whole brain analysis for the effect of perception reveals two regions that are significantly modulated by perceptual state (p < .001 uncorrected). Both right intraparietal sulcus (rIPS) and the precuneus show greater activity levels during split percept relative to the grouped percept.

An exploratory analysis of the whole brain reveals voxels in right intra-parietal sulcus (rIPS) and the precuneus which surpass a threshold of p < .001 (uncorrected) for an effect of perceptual state (Figure 4). The region in rIPS is 3-5 cm anterior to the two regions found by Cusack (2005) to be sensitive to perceptual state. In contrast, perceptual sensitivity has not been reported in the precuneus region before. A list of the MNI coordinates and effect sizes of cluster maxima can be found on Table 1. In order to directly asses if our data were consistent with the previous findings, we analyzed our smoothed data (8 mm FWHM) at both sets of MNI coordinates reported by Cusack. The posterior IPS region (MNI coordinates: 34, -72, 38) showed a trend level effect of percept (p = 0.076), while the anterior region (MNI coordinates: 44, -48, 48) showed a significant effect of percept (p = 0.038). All parietal regions discussed in this section showed a negative beta, indicating that the split percept produces a higher level of activity than the grouped percept. The sign of this relationship is also consistent with Cusack's findings. Unlike auditory cortex, an analysis of the patterns of these parietal activations yielded no significant correlation (p = 0.377) between the betas for acoustic response and perceptual state.

**Table 1:** Clusters of voxels significantly modulated by percept

Hemisphere	Anatomical Region	Cluster Size (Voxel)	Peak MNI Coordinates	Peak T-Score
**R**	Intraparietal Sulcus	36	26 -32 67	5.45
	Precuneus	49	-1 -59 46	5.29

## Discussion

Our results show that early AC reflects the sustained perceptual phenomenon of streaming, and that the spatial patterns of AC subregions most sensitive to sound also show the greatest perceptual effect. This broadly supports the theory that sound segregation modulates the same neural circuits that process basic sound object features [18]. The direction of our perceptual effect, that AC activity is greater for separate streams than a grouped stream, and the existence of buildup in streaming supports the notion that the grouped percept is the default perception and that additional metabolic effort occurs when perceiving two or more streams. Animal models of streaming propose that this effort manifests as narrowing receptive fields of neurons sensitive to the stimulus dimension along which the putative streams are separated [14]. While there are examples where the local circuits of a cortical region can promote sharpening of receptive fields through short-range reciprocal inhibition from audition [32], vision [33], and olfaction [34], an alternative explanation is that other cortical regions could direct or otherwise interact with local streaming related sharpening. Theories of auditory streaming have so far not made specific claims about whether this modulation arises from local network processes or interaction between AC and higher level areas.

Our data suggests that AC is only one of several regions involved in streaming. Higher cortices may play a cooperative role by interpreting a segregated scene or modulating the streaming itself. For instance, consistent with Cusack's previous work [23], rIPS may track the number of distinct objects after they have been segregated by auditory cortex or it may allow broad behavioral goals to influence streaming mechanisms. Such high level control of auditory streaming mechanisms is evident behaviorally as listeners can consciously influence the number of perceived objects [12]. Further studies that are optimized to detect functional connectivity between multiple cortical regions and explicitly modulate top down signals such as task demands or expectation may shed light on the interaction between higher level areas and AC.

Technical challenges may have played a key role in the prior lack of fMRI based evidence for AC's involvement in the continuous maintenance of auditory streaming. Cusack's study used sparse scanning techniques, and a number of stimuli that spent a large amount of time in a single percept [23]. Both of these factors would drastically reduce statistical power, possibly giving a false negative for the effects of perceptual state. In addition, general linear models (the usual method employed for analyzing fMRI data sets) can have difficulty dissociating the activity for bi-stable percepts. If regressors are included for each perceptual state independently, the alternating nature of bi-stable percepts can cause the regressors to become collinear after standard filtering needed to remove known low frequency noise in fMRI data. We instead used a single bimodal perceptual regressor that, although precluding analyses on each percept alone, offers a powerful measure of the activity distinguishing between percepts.

It is also worth considering why EEG and MEG studies have not found effects outside of auditory cortex. Clearly, models of streaming would benefit from data on non-AC (e.g. rIPS) neural signals with the excellent temporal resolution of EEG and MEG. One possibility is that higher level processes lack consistent phase-locking with the stimuli. So while previous studies have focused on stimulus-locked event related potentials/fields, induced activity (loosely time-locked but not precisely phase-locked) may have remained undetected. In addition, the MEG studies cited used source-filtered waveforms that ignored currents generated outside of AC [21,22]. Designs and analyses that detect non-stimulus locked activity and integrate data from multiple current sources may improve the correspondence between findings in these different modalities.

Surprisingly in our attentional control task, subjects were no worse (or better) at detecting targets while streaming separate objects. This has direct relevance for theories of how attention operates within, or spreads across, object representations [35-37]. Our results suggest that there is no cost associated with small numbers of objects. In general, auditory streaming appears to be a relatively untapped paradigm for the study of object-based attention considering the advantages of having a single controlled stimulus which fluctuates between two different object schema every few seconds. Some investigations have suggested that certain task sets, such as deviant detection in a single stream, may promote perceptual segregation [38]. Even though the recorded d' values between our two perceptual states is equivalent, it may be that performing a secondary task designed to spread attention across multiple streams may have impacted participants' overall streaming percentage. However, this contextual effect should not influence the interpretation of our results because our calibration procedure ensures that we find a streaming threshold in the presence of both simultaneous tasks and such task demands are equally present during both split and grouped percepts. Thus any comparison between the two percepts controls for task related factors.

## Conclusions

Using carefully calibrated stimuli and a focused approach, we are the first group to show sustained activity in AC that distinguishes between perceptual streaming states. Auditory cortex showed higher levels of activity for split percept compared to the grouped percept. These results strengthen the continuity between multiple lines of evidence supporting a role for AC in the formation and maintenance of auditory streams. At the same time, our work is consistent with previous fMRI experiments to suggest that AC does not function alone in this task. Future studies will address the interaction between multiple cortical regions and improve our understanding of this important perceptual phenomenon.

## Authors' contributions

KH designed the study, collected data, performed the analyses and drafted the manuscript. CB participated in the design of the study, analysis selection, collection of data and editing of the manuscript. DY participated in data collection and reviewed the manuscript. LM participated in study design and coordination and edited the manuscript. All authors read and approved the final manuscript.
